# Neuropharmacology of language: ERP evidence for catecholaminergic modulation of syntactic processing

**DOI:** 10.1073/pnas.2606806123

**Published:** 2026-10-22

**Authors:** Yingying Tan, Ashley G. Lewis, Iris C. Schmits, Robert-Jan Verkes, Roshan Cools, Peter Hagoort

**Affiliations:** ^a^ Shanghai International Studies University, Institute of Language Sciences, Shanghai 201620, China; ^b^ Max Planck Institute for Psycholinguistics, Neurobiology of Language Department, Nijmegen 6525 XD, the Netherlands; ^c^ City University of Hong Kong, Department of Linguistics and Translation, Kowloon, Hong Kong Special Administrative Region, China; ^d^ Radboud University, Donders Institute for Brain, Cognition and Behaviour, Center for Cognitive Neuroimaging, Nijmegen 6525 EN, the Netherlands; ^e^ Radboud University Medical Center, Department of Psychiatry, Nijmegen 6525 GA, the Netherlands

**Keywords:** catecholamine, syntactic unification, methylphenidate, garden-path sentence, N400/P600

## Abstract

A few studies have shown that brain catecholamines are implicated in language comprehension. However, the specific cognitive processes they modulate remain underexplored. We hypothesized that catecholamines enhance the unification of syntactic roles and word meanings into coherent multiword structures. This study combined electroencephalography and psychopharmacology to examine the effects of a catecholaminergic stimulant (methylphenidate) on processing sentences containing local syntactic ambiguities. Methylphenidate amplified the P600 event-related potential effect, which has been linked to the recruitment of cognitive resources for processing sentences with increased syntactic load. The drug’s impact on semantic processing was subtler, showing a suggestive attenuation of the N400 effect to ambiguous sentences. Furthermore, the increase in the P600 effect under methylphenidate was most pronounced in participants with lower working memory capacity. This study provides evidence for linking catecholamine activity to syntactic unification in healthy individuals. Elevated catecholamine levels appear to increase the cognitive effort invested in syntactic unification, and to support the dynamic prioritization of syntactic information when contextual demands require it.

## Significance

How the brain’s neuromodulatory systems influence language, a defining human capacity, remains poorly understood. Here we show that catecholaminergic neurotransmission—primarily involving dopamine and noradrenaline—acts as a neurobiological relevance signal during sentence comprehension, dynamically tuning the cognitive effort devoted to processing grammatical structure. Pharmacological elevation of catecholamine levels enhanced the brain’s electrophysiological response to syntactically ambiguous sentences, suggesting greater recruitment of cognitive resources for syntactic unification. This effect was most pronounced in individuals with lower baseline working memory capacity, consistent with the view that catecholaminergic gain preferentially benefits those with suboptimal baseline performance. By linking catecholamines to syntax—a core property of human language—this study reveals how neuromodulatory signaling flexibly prioritizes linguistic processing in response to contextual demands.

Producing and understanding language are among the most complex cognitive functions that humans command. From the brain perspective, areas in frontal and temporoparietal cortex have long been known to be critically involved. In addition, event-related potential (ERP) effects such as the N400 and the P600 are well-established electrophysiological markers of language processing. Despite a wealth of fMRI and ERP studies on the neural architecture of language processing, hardly anything is known about the influence of the chemical neuromodulators, like dopamine and noradrenaline, on language processing. Unraveling the role of these neuromodulators is important, as they modulate cognition, and are key ingredients of widely used cognitive enhancers such as methylphenidate. Although such drugs are known to improve language comprehension, the mechanisms by which they act remain unknown. The present study addresses the neuromodulation of language by examining a key aspect of human language, namely the processing of syntax. Below we will first discuss what is known thus far about the neuromodulation of language.

The catecholaminergic neuromodulators dopamine and noradrenaline are both among the brain’s major neuromodulatory systems. They innervate multiple, relatively segregated fronto-striatal pathways that connect the prefrontal cortex (PFC) with the basal ganglia (BG), while also projecting widely to other brain regions, including the parietal cortex and the amygdala (1). Catecholamines play a fundamental role in regulating various PFC-based cognitive functions, such as attention, working memory, and language (2, 3).

The very few extant neuromodulation studies investigating language processing have highlighted a crucial role of the catecholamines, particularly in semantic processing. Early evidence emerged from observations that patients with catecholamine-related dysfunctions, like those with Parkinson’s disease (PD), often exhibit language deficits in semantic and pragmatic processing (4, 5). More recent studies have expanded our understanding of the catecholamine system’s role in semantic processing in healthy populations, using psychopharmacological and neuroimaging methods. These studies have shown that administering catecholaminergic stimulants such as methylphenidate (MPH) and levodopa modulates priming of semantically related word pairs (e.g., bank-river) (6–8).

Tan and Hagoort (9) further demonstrated an important role for catecholamines during sentence processing (9). In their study, participants read semantically congruent or incongruent sentences following administration of 20-mg MPH or a placebo. In one condition, participants read for comprehension; while in the other, they attended only to the font size, a task for which the processing of sentence meaning is irrelevant. Results showed that semantically anomalous words in the sentences elicited a larger N400 ERP amplitude. The N400 is a negative going voltage deflection occurring approximately 250 to 500 ms after word onset, with a peak at around 400 ms. The amplitude of the N400 was proportional to the degree of semantic processing difficulty (10, 11). Notably, the effect of catecholamine modulation was task-dependent. Elevated catecholamine levels increased participants’ sensitivity to semantic anomalies in the task-irrelevant condition, evidenced by a larger N400 amplitude to semantically incongruent sentences. These findings suggest that catecholamines enhance semantic unification even when semantic processing is not the primary task goal, presumably by increasing the saliency of combinatorial processing whenever words can potentially be combined to get sentence-level meaning.

While previous studies have provided some insights into the role of catecholamines in semantic processing, their contribution to another central aspect of language processing, namely syntactic unification, is still unknown. Syntactic unification refers to the procedures combining syntactic parts of speech (e.g., nouns, verbs, adjectives) into grammatically well-formed sequences. Broca’s region has been found to be critically involved (12). Importantly, neuropsychological and neuroimaging studies have also linked the basal ganglia (BG) to syntactic unification. The BG are heavily innervated by dopaminergic projections from the substantia nigra and noradrenergic projections from the locus coeruleus (13), both essential for BG modulation. Syntactic unification is impaired in patients with BG deficits (14). For instance, PD patients struggle with syntactically complex sentences (15–17). Additionally, neuroimaging evidence demonstrates BG activation during syntactic unification (18–20). We hypothesize that elevated catecholamine levels enhance syntactic unification through fronto-striatal pathways. Investigating the role of catecholamines in syntactic computation is crucial for understanding the neurobiological mechanisms underlying language processing. This line of research will advance our understanding of the “binding problem” in language, which concerns how individual words are dynamically unified into multiword utterances (21).

In this study, we examined the impact of catecholamine levels on syntactic unification using a classical garden-path paradigm (22), combining psychopharmacological manipulation with the recording of ERPs. In the course of normal language processing readers are often confronted with temporary ambiguities in sentence structure that only get resolved further downstream in the sentence. We exploited the presence of these so-called syntactic ambiguities in our materials. As shown in Table 1, in the ambiguous condition readers tend to initially interpret “guitarist” as being grouped with the preceding noun “drummer.” However, upon encountering the disambiguating word (“packed”), they have to revise their initial interpretation to understand that “guitarist” starts a new clause and cannot be grouped together with “drummer” as part of one noun phrase. In contrast, in the unambiguous condition from Table 1, the comma after “drummer” indicates a clear phrase boundary, guiding readers to correctly interpret the sentence as involving two clauses from the start. This prevents any need for reanalysis at “packed,” since the parser is never led down the garden path in the first place. Additionally, we included a filler condition composed of sentences with different syntactic structures to make the ambiguous sentences less prominent/frequent and to discourage participants from strategically anticipating the ambiguities. Comparison between ambiguous and unambiguous conditions at the disambiguating word has been shown to reliably isolate effects related to resolving local structural ambiguity during sentence processing, as indexed by a P600 effect in the EEG recordings (22, 23).

**Table 1.** Example materials for the experimental sentences and their English translation (in italics)

Condition	Sentence
Ambiguous	De fan belaagde de drummer en de gitarist **pakte** de instrumenten in.
	*The fan attacked the drummer and the guitarist packed the instruments up*.
Unambiguous	De fan belaagde de drummer, en de gitarist **pakte** de instrumenten in.
	*The fan attacked the drummer, and the guitarist packed the instruments up*.
Filler	De fan belaagde de drummer en de gitarist omdat de muziek te hard was.
	*The fan attacked the drummer and the guitarist because the music was too loud.*

The comma after the second noun (drummer in the example) removes the syntactic ambiguity.

The P600 is a positive going voltage deflection observed roughly between 500 and 900 ms, traditionally associated with syntactic processing difficulties (24, 25). Its functional interpretation remains debated. Some accounts emphasize syntactic reanalysis (26, 27), others interpret the P600 as a continuous index of integration effort that scales with the cost of updating the unfolding sentence representation (28, 29), and still others link the component to the broader P3b family of late positivities (30). What these views share is the proposal that the P600 reflects increased cognitive effort when an incoming word is costly to integrate, rather than a passive byproduct of processing difficulty. Whether this effortful recruitment of resources is regulated by catecholaminergic systems is, to our knowledge, an open question.

Lesion-ERP evidence suggests that catecholaminergic systems do modulate the P600. Basal ganglia damage selectively attenuates the P600 while sparing the P3b (31, 32), implicating fronto-striatal circuitry in the P600 effect. The basal ganglia in turn receive dense dopaminergic and noradrenergic projections that modulate fronto-striatal information processing (1, 13), making catecholamines a natural candidate for regulating the P600 effect. Catecholamines have also been linked to cost–benefit evaluation of cognitive effort (33–36). We hypothesized that elevated catecholamine levels increase cognitive effort invested when sentence processing becomes more demanding, thereby modulating the P600 amplitude observed during garden-path processing.

The present study provides evidence that the catecholamine system modulates the neural response underlying syntactic unification. MPH administration robustly enhanced the P600 effect and marginally attenuated the N400 response to syntactically ambiguous sentences compared with placebo, with no comparable changes for unambiguous sentences. These results highlight the specific role of elevated catecholamine levels in prioritizing syntactic unification during ambiguity resolution. We interpret the P600 enhancement as reflecting increased recruitment of task-relevant resources, consistent with the view that elevated catecholamine levels serve as a relevance signal that boosts the cognitive resources allocated to syntactic unification. Notably, exploratory analyses showed that the P600 enhancement was most pronounced in participants with lower baseline working memory (WM) capacity, in line with the proposal that lower-span individuals have greater room for pharmacological gain (2, 3, 33, 34, 36, 37). Our study advances the understanding of how catecholamines influence language processing by showing that their impact extends beyond semantics during sentence comprehension (9). We show that syntax, often seen by linguists as the unique feature of human language, can be up- or down-regulated by catecholaminergic manipulation.

## 1. Results

### 1.1. Catecholamines Enhance Syntactic Unification in Sentence Comprehension: ERP Evidence.

**Fig. 1.**
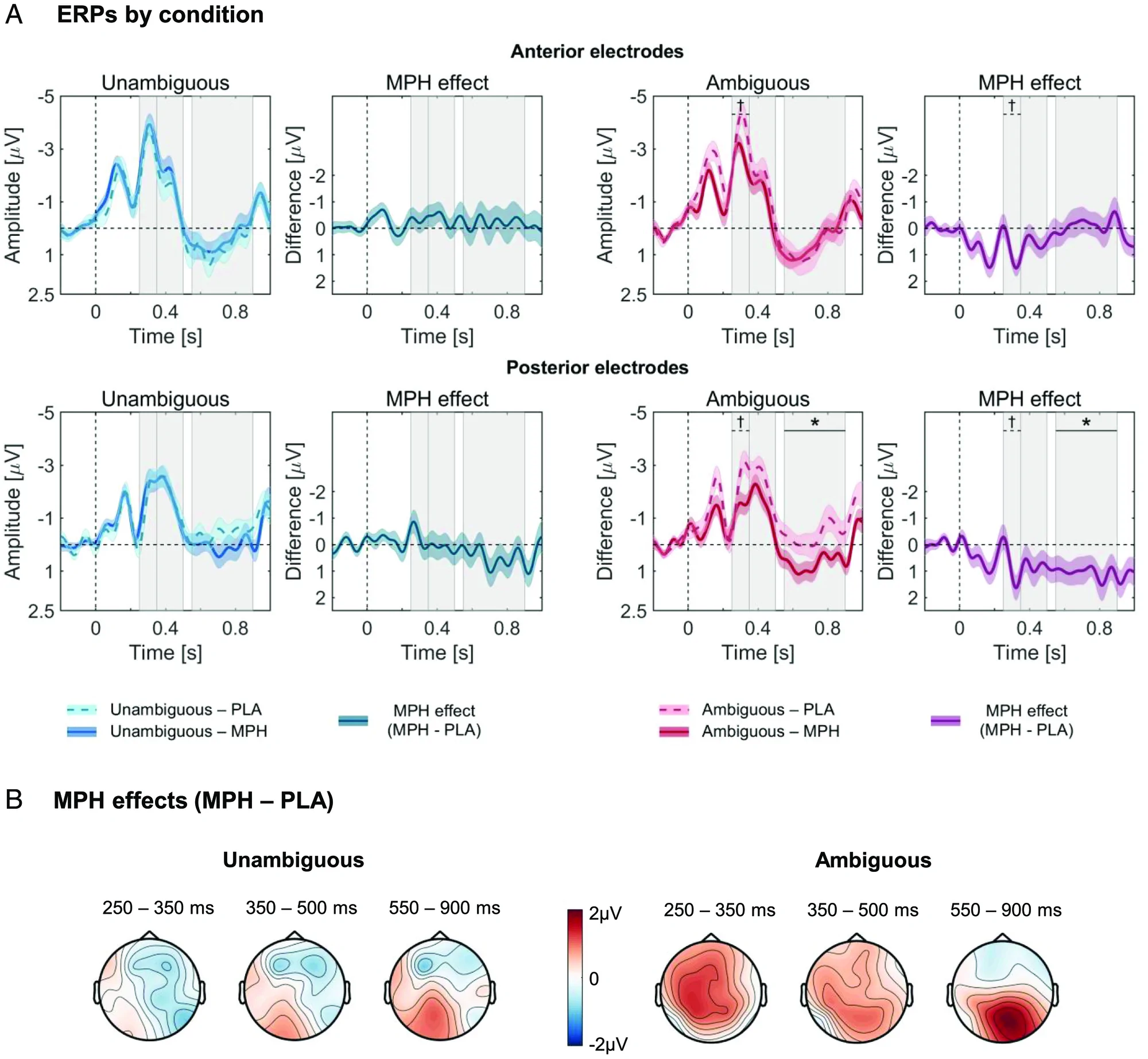
Methylphenidate administration modulated neural activity during sentence comprehension, enhancing syntactic unification-related P600 responses (550 to 900 ms) for ambiguous sentences, with suggestive evidence for a reduction in early N400 amplitudes (250 to 350 ms) in the same condition. (*A*) Grand-averaged ERPs at anterior (F3/4, FC1/2, F7/8, FC3, Fz, FCz) and posterior (CP1/2, CP5/6, P3/4, P7/8, Cz, Pz) electrode clusters. Waveforms are shown separately for the methylphenidate (MPH) and placebo (PLA) treatments within the unambiguous and ambiguous sentences. The columns labeled “MPH effect” show the corresponding difference waves (MPH−PLA) at the same electrode clusters, reflecting the pharmacological effect of methylphenidate on the ERP response. Negativity is plotted upward. Shaded regions indicate the SEM across participants. For illustrative purposes only, a 10-Hz low-pass filter and a baseline correction from −200 to 0 ms relative to critical word onset were applied. Reliable evidence from Bayesian linear mixed-effects models is marked with asterisks (*); suggestive evidence is marked with daggers (†). (*B*) Scalp topographies of the MPH effect (MPH−PLA) for the unambiguous and ambiguous conditions, averaged across each time window.

As shown in Fig. 1, the critical word (i.e., the disambiguating word of the garden-path sentence; “packed” in the example sentences in Table 1) elicited a classic ERP pattern in response to visually presented verbal stimuli. There was an N1-P2 complex in the first 200 ms postword onset in all conditions, followed by a biphasic N400/P600 pattern to ambiguous sentences. Our primary aim was to examine the impact of the drug on syntactic ambiguity resolution, described in detail below.

Table 2 provides a summary of the behavioral and ERP effects reported in this section. Mean EEG amplitudes extracted on a single-trial basis from three predefined time windows were analyzed using Bayesian linear mixed-effects regression models (38). We treat a predictor as providing reliable evidence when the 95% credible interval (CrI) of its posterior estimate excluded zero, and as providing suggestive evidence when the 90% CrI excluded zero. Full model specifications and complete statistical results are provided in *Materials and Methods* and *SI Appendix*, Fig. S1 and Table S1.

**Table 2.** Summary of MPH effects on ERP and behavioral measures during garden-path sentence comprehension

Measure	Effect	Mean [95% CrI]	Evidence
*Prespecified ERP effects (critical word, e.g., “packed”)*			
P600	Ambiguous: MPH > Placebo (posterior)	0.03 [0.00, 0.05]	^*^
	Unambiguous: n.s.	−0.01 [−0.03, 0.02]	n.s.
N400	Ambiguous: MPH < Placebo (lateral)	0.04 [−0.02, 0.10]	^†^
	Unambiguous: n.s.	−0.02 [−0.08, 0.04]	n.s.
*Behavioral effects (comprehension question)*			
RT	Drug effect (MPH faster)	−0.08 [−0.13, −0.02]	^*^
Accuracy	Ambiguous: MPH > Placebo	0.17 [−0.04, 0.38]	^†^
*Exploratory analyses: influence of working memory*			
Placebo	*P600 × WM*	0.03 [0.00, 0.07]	^*^
MPH	*P600 × WM*	0.02 [−0.02, 0.06]	n.s.
*Control analyses: CPS effect (2nd noun, e.g., “drummer”)*			
CPS	Drug effects	0.01 [−0.02, 0.05]	n.s.

Note. Posterior means and 95% Crls from Bayesian linear mixed-effects models.

^*^Reliable: 95% CrI excludes zero (prespecified criterion).

^†^Suggestive: ≥90% of posterior probability mass on one side of zero. n.s.: no credible effect. Prespecified contrasts are reported under “Prespecified ERP effects”; analyses identified after data collection or motivated by visual inspection of the waveforms are reported under “Exploratory analyses.” See *Materials and Methods* for the full model specifications and *SI Appendix*, *Supplementary Materials* for full posterior summaries and prior-sensitivity checks.

#### 1.1.1. P600 (550 to 900 ms).

MPH administration provided reliable evidence for enhanced syntactic unification–related activity in the P600 time window compared with placebo. Specifically, MPH increased the late positive deflection only when the critical word was embedded within structurally ambiguous sentences, but exerted no measurable effect when sentences were structurally unambiguous (Fig. 1). There was reliable evidence for a three-way interaction between Drug, Ambiguity, and Anteriority at both lateral (mean = 0.01, 95% CrI [0.00, 0.02]) and midline electrodes (mean = 0.01, 95% CrI [0.00, 0.01]). Planned post hoc analyses revealed an interaction between Drug and Ambiguity, only over posterior electrodes (lateral posterior electrodes: mean = 0.02, 95% CrI [0.00, 0.03]; midline posterior electrodes: mean = 0.02, 95% CrI [0.00, 0.03]). Further analysis showed that critical words in ambiguous sentences elicited a more positive deflection following MPH administration compared with placebo (lateral posterior electrodes: mean = 0.03, 95% CrI [0.00, 0.05]; midline posterior electrodes: mean = 0.02, 95% CrI [0.00, 0.05]). The main effect of Drug did not reach our threshold for reliable or suggestive evidence, and there was no evidence for any interactions between drug effects and the Drug Order predictor (designed to account for whether participants were administered the drug or placebo in the first or second EEG recording session).

#### 1.1.2. N400 early (250 to 350 ms).

MPH administration led to suggestive evidence for a reduced N400 effect between 250 to 350 ms compared with placebo. This reduction was specific to the Ambiguous condition, as no reliable or suggestive evidence for a drug effect was observed in the Unambiguous condition (Fig. 1). There was suggestive evidence for an interaction between Drug and Ambiguity at the lateral electrodes (mean = −0.03, 95% CrI [−0.06, 0.01], with 95.2% of the probability mass indicating a negative effect), suggesting a trend toward a reduction in the N400 effect following MPH administration. Planned post hoc analyses revealed a numerical trend toward smaller N400 amplitudes in the Ambiguous condition under MPH compared with placebo at lateral electrodes (mean = 0.04, 95% CrI [−0.02, 0.10], with 90.0% of the probability mass indicating a positive effect). No comparable effect was observed in the Unambiguous condition. The Drug Order predictor did not reach our threshold for reliable or suggestive evidence and did not interact with any other drug-related predictors.

#### 1.1.3. N400 late (350 to 500 ms).

There was no evidence for either drug-related or ambiguity-related effects in this time window.

### 1.2. Exploratory Analysis: Interaction Between MPH’s Effects and WM Capacity.

**Fig. 2.**
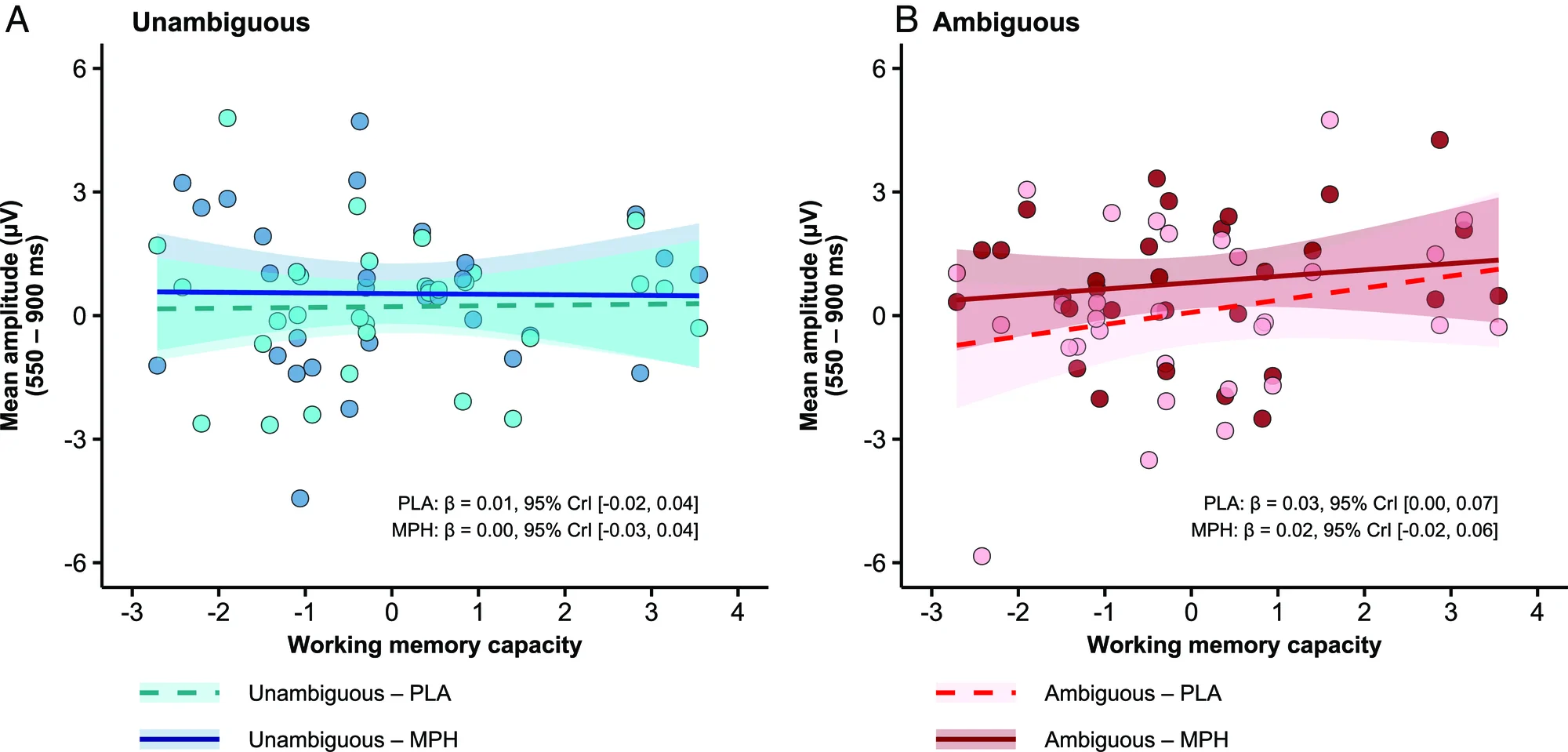
Baseline working memory capacity modulates the effect of methylphenidate (MPH) on neural responses to ambiguous sentences, as observed at posterior electrodes (550 to 900 ms). Scatter plots with regression lines show the relationship between mean ERP amplitude at posterior electrodes in the P600 time window and standardized working memory scores in the unambiguous (*A*) and ambiguous (*B*) sentence conditions, for both placebo (PLA) and MPH. Each point corresponds to an individual participant’s data. The shaded areas represent the 95% CI. Trial-level mean amplitudes were modeled using Bayesian linear mixed-effects regression, with working memory score, sentence ambiguity, drug condition, and electrode anteriority included as fixed effects together with their interactions.

To investigate whether the effects of MPH on syntactic unification depend on individuals’ baseline WM capacity, we conducted an exploratory analysis. Our results showed that MPH modulates syntactic unification in a manner influenced by WM capacity. Specifically, there was reliable evidence for a four-way interaction between Drug, Ambiguity, Anteriority, and WM in the P600 time window at both lateral (mean = −0.01, 95% CrI [−0.01, 0.00]) and midline electrodes (mean = 0.00, 95% CrI [−0.01, 0.00]). Planned post hoc analyses further revealed reliable evidence for a three-way interaction between Ambiguity, WM, and Anteriority following placebo administration (mean = 0.02, 95% CrI [0.01, 0.03]), which was absent following MPH administration. As shown in Fig. 2*A*, following placebo administration participants with lower WM capacity displayed a reduced positive deflection in response to ambiguous sentences at posterior electrodes compared with participants with higher WM capacity (mean = 0.03, 95% CrI [0.00, 0.07]). In contrast, this WM-related difference was no longer evident following MPH administration. This finding aligns with prior research indicating that the magnitude of the P600 effect is positively correlated with individuals’ WM capacity in the absence of pharmacological intervention (39). More importantly, these results extended existing psychopharmacological evidence (3) by demonstrating that the effects of catecholaminergic drugs are (at least partially) dependent on baseline WM capacity. Specifically, participants with lower WM capacity showed larger MPH-related changes in P600 effect than higher-WM capacity individuals, who exhibited relatively little change. This pattern is consistent with the notion that lower-span individuals have a greater dynamic range for modulation (3), with larger drug-related changes reflecting greater room for modulation before reaching a functional ceiling. Full statistical model outputs are available in *SI Appendix*, Table S2.

### 1.3. The Closure Positive Shift (CPS).

As a control analysis, we also examined ERPs time-locked to the second noun (e.g., “drummer”) that was presented either with or without a comma (*SI Appendix*, Fig. S2). This position can, in principle, elicit a CPS, an ERP marker of prosodic or structural boundary detection, when comparing conditions with or without a comma (Unambiguous vs. Ambiguous condition) (40). We found no evidence of drug-related effects at this noun position, suggesting that the MPH effects observed at the critical word (e.g., “packed”) cannot be attributed solely to overall enhanced arousal or attention following MPH administration. Moreover, despite the absence of a CPS effect, our observation of processing differences between unambiguous and ambiguous sentences at the disambiguating verb in both the early N400 and the P600 time windows suggests that participants nevertheless utilized these punctuation cues for structural interpretation at later time points in the sentence. Full statistical model outputs are available in *SI Appendix*, Table S3.

### 1.4. Behavioral and Physiological Effects of Methylphenidate.

Accuracy and reaction time data for the comprehension questions that followed each sentence (*Materials and Methods*) are reported in *SI Appendix*, Fig. S3 and Table S4. Participants were generally attentive and accurate during the experiment (Mean accuracy = 85.0%, SD = 10.4%, range: 70.8 to 96.7%). Relative to placebo, methylphenidate reliably reduced response times to the comprehension questions (mean = −0.08, 95% CrI [−0.13, −0.02]), and there was suggestive evidence that it increased accuracy in the Ambiguous condition relative to placebo (mean = 0.17, 95% CrI [−0.04, 0.38], with 94.8% of the probability mass indicating a positive effect). Full statistical model outputs are available in *SI Appendix*, Fig. S4 and Table S5.

Participants’ cardiovascular responses, subjective feelings (BL-VAS ratings), sEBR, and all related analyses are presented in *SI Appendix*, Table S6. These measurements were all comparable before drug administration. Following MPH administration, participants exhibited increased heart rate [*t*(29) = −6.40, *P* < 0.001] and systolic blood pressure [*t*(29) = −4.32, *P* < 0.001] compared with the placebo condition. They also subjectively reported heightened alertness and reduced calmness in the BL-VAS ratings. These results help verify that drug administration did indeed result in heightened systemic and CNS drug levels (9, 34, 41, 42). There was no statistically significant change in participants’ sEBR following MPH administration compared with placebo.

## 2. Discussion

The present psychopharmacological and EEG study provides evidence that catecholamines modulate the neural correlates of syntactic unification in healthy individuals. Elevated catecholamine levels have been proposed to enhance participants’ ability to prioritize task-relevant information, which in this study was the syntactic information, enabling cognitive resources to be dynamically allocated according to ongoing processing demands (43). Our findings extend this account from the semantic domain (9) to syntactic processing, and further suggest that the magnitude of catecholaminergic gain may depend on baseline working memory capacity.

The key finding of this study is that elevated catecholamine levels during garden-path sentence comprehension influenced neural signatures sensitive to syntactic processing. Specifically, we observed a pronounced increase in the P600 amplitude when participants read syntactically ambiguous sentences under MPH administration compared with placebo. The garden-path paradigm provides a particularly clean window onto the processing of syntactic ambiguity. The disambiguating word in our materials carried no semantic anomaly, no contextual implausibility, and no lexical surprisal beyond what was matched in the unambiguous control. The feature that differed across conditions was the presence of syntactic ambiguity, which engages additional processing at the disambiguating word relative to the unambiguous control. We therefore interpret the modulation of the P600 by MPH as a modulation of the processing engaged by syntactic ambiguity. The P600 effect has traditionally been linked to the late, controlled phase of syntactic processing, including the reanalysis of sentence structure when initial interpretations fail (24, 25). It has also been linked to increased recruitment of cognitive resources to resolve syntactic processing difficulty (28, 29). Therefore, we interpret the larger P600 under MPH as reflecting a catecholaminergic boost of cognitive effort during the resolution of syntactic ambiguity. While a larger P600 effect reflects greater neural investment, MPH appears to upregulate the value participants (implicitly) assign to engaging in demanding cognitive tasks (44). The enhanced P600 may therefore represent a shift in the allocation of cognitive resources toward syntactic unification, with MPH mitigating the subjective costs that might otherwise favor shallower processing under placebo.

Existing neuropsychological and neuroimaging studies implicate the BG and fronto-striatal connectivity in syntactic processing (14, 15, 18, 19), making this circuitry a plausible substrate for the catecholaminergic modulation of syntactic unification observed here. MPH acts by blocking the DA and NA transporters, and the DA transporter is particularly abundant in the BG. We therefore propose that the MPH effects reported here reflect, at least in part, catecholaminergic modulation of fronto-striatal connectivity, with altered BG outflow influencing syntactic unification in the PFC and Broca’s region. However, MPH also increases catecholamine levels directly in the PFC by blocking DA and NA transporters there (45), and a direct cortical contribution to the observed effects therefore cannot be ruled out based on the current data. Furthermore, the present design cannot adjudicate between dopaminergic and noradrenergic contributions to syntactic unification. Although DA has been proposed to play a central role in regulating language processing (5), some accounts link the P600 effect to the phasic response of the locus coeruleus-NA (LC-NA) system, given its possible relationship to the P3b ERP component (30). The P3b is itself modulated by the LC-NA system and shows larger amplitude in response to familiar but infrequent task-relevant stimuli (46). Pharmacological dissociations using more selective compounds, or designs exploiting individual differences in baseline DA and NA function, will be needed to disentangle these pathways.

The N400 between 250 and 500 ms was attenuated under MPH administration compared with placebo, although the evidence for this effect was suggestive. The attenuation was driven by a reduced N400 response to ambiguous sentences, with no comparable drug effect for unambiguous sentences. The N400 component is a well-established neural marker of semantic processing, being less negative when words undergo less elaborate semantic processing (11, 47). We hypothesize that the attenuated N400 amplitude to ambiguous sentences reflects a relative prioritization of syntactic over semantic unification in cases of increased syntactic processing demands when MPH is administered. We offer this as a working hypothesis rather than a firm conclusion, because the present design did not orthogonally manipulate semantic anomaly. Distinguishing these accounts will require designs that cross syntactic and semantic processing difficulties within the same experiment.

Comparison of the current results with our previous study on MPH’s influence on semantic anomaly (9) is informative. In both experiments, MPH increased the amplitude of a late positivity when participants read sentences for comprehension, indicating that elevated catecholamine levels facilitated unification regardless of whether the increased processing load is semantic or syntactic in nature. However, MPH had opposing effects on the early N400 response in sentence comprehension: it increased N400 amplitude for semantically congruent critical words in our previous study (9), but reduced it for syntactically ambiguous critical words in the present study. One possible explanation is that, under MPH, participants flexibly adjust their processing goals to prioritize semantic or syntactic unification depending on contextual demands. In the current study, syntactic ambiguity was the most salient feature, prompting participants to allocate additional cognitive resources to syntactic unification, as evidenced by the enhanced P600 effect. This shift came at the expense of semantic-level processing, as evidenced by the suggestive reduction in N400 amplitude. In contrast, the sentence materials in our previous study contained only a semantic-anomaly manipulation, making the semantic level the most salient linguistic feature and accordingly eliciting a greater N400 response. The contrasting MPH effects on the N400 between our previous and current experiments provide preliminary support for the hypothesis that elevated catecholamine levels may selectively prioritize task-relevant language processing (9), with a focus on either semantic or syntactic unification depending on the context. This hypothesis aligns with previous research showing that elevated catecholamine levels enhance cognitive processing in the PFC by flexibly gating task-relevant or salient processing via fronto-striatal pathways (1, 35, 43, 48, 49). This account is best treated as a hypothesis to be followed up in future work, given that processing goals were not directly manipulated in our study.

We also observed that the MPH-related increase in the P600 effect was most pronounced in participants with lower baseline working memory capacity. This finding aligns with our previous observation that lower-span participants showed larger N400 reductions for semantic anomalies under MPH (9), and with the broader literature on baseline-dependent catecholaminergic effects (3, 33, 34, 36, 37). Under placebo, lower-span individuals may engage in shallower processing to avoid the cognitive cost of reanalysis. MPH likely shifts the subjective weighting of these costs against the benefits of reanalysis. This shift may make low-span individuals more amenable to engaging reanalysis under comparable conditions of cognitive demand, and enable more robust recruitment of resources that manifests as P600 enhancement. Lower-span individuals may therefore exhibit a greater dynamic range for pharmacological modulation, whereas higher-span participants may already operate near a functional ceiling (3). On this view, the larger P600 reflects a drug-induced increase in cognitive effort invested in syntactic unification. This may naturally lead one to ask a further question about the underlying mechanism. Given the well-established link between WM and syntactic unification (50) and between WM and dopamine (2, 3), catecholamines may either exert a direct influence on the neural correlates of language processing, or an indirect influence by modulating WM capacity. Disentangling these possibilities is an important goal for future research, which will require designs that can dissociate drug effects on syntactic processing from drug effects on working memory itself.

In summary, we found a neuropharmacological effect on syntactic processing. Catecholamines upregulate sentence processing when syntactic unification becomes more demanding, as reflected in the amplitude of the P600. This effect is modulated by participants’ WM capacity, with a stronger drug effect for participants with a lower WM capacity. This study provides evidence for the neuromodulation of key processes in language comprehension. Although the field of neuropharmacolinguistics is still in its infancy, it is clear that it has a role to play in understanding the neurobiological basis of our uniquely human capacity for language.

## 3. Materials and Methods

### 3.1. Participants.

Forty native speakers of Dutch took part in the study (mean age = 22.6 y, SD = 3.2, range: 18 to 32; 20 men). Four participants dropped out before completion of the study, either because of personal reasons (N = 2), or due to drug expiration (N = 2) during the COVID-19 lock down period. Therefore, data were available for a total of 36 participants (mean age = 22.9 y, SD = 3.1, range: 18 to 32; 18 men). The study was conducted according to Good Clinical Practice (GCP) guidelines and was approved by the regional ethics committee in the Netherlands (“Commissie Mensgebonden Onderzoek,” CMO region Arnhem-Nijmegen, The Netherlands: protocol NL69700.091.19).

Before starting the experiment, all participants provided written informed consent to take part in the study, and were extensively screened by a responsible physician from Radboud University Medical Center. Participant exclusion criteria were as follows: any current or previous neurological, psychiatric, or language disorders; clinically significant hepatic, cardiac, renal, metabolic, or pulmonary disease; epilepsy; habituated smoking; glaucoma; irregular sleep/wake rhythm; possible pregnancy; having first-degree family members with a current or previously diagnosed psychiatric disorder. Participants were required to abstain from alcohol and smoking for 24 h and from psychotropic medication or recreational drugs for 72 h prior to each experimental session. In the morning of each EEG testing day, participants were asked to eat a light breakfast without any caffeinated drink.

Five participants were excluded from the statistical analyses due to excessive artifacts in the EEG recordings. An additional participant was excluded because of low accuracy in the sentence comprehension experiment (52%). Therefore, 30 participants were included in the final data analyses (16 men; mean age = 22.2 y, SD = 2.5, range: 18 to 30), all were right-handed and had normal or corrected-to-normal vision.

### 3.2. Pharmacological Design.

The study employed a double-blind, placebo-controlled, randomized, cross-over, within-participant design (ClinicalTrials.gov ID: NCT05272397). Each participant was tested in three sessions. The first session served as an intake, during which participants were screened based on inclusion/exclusion criteria and several individual differences measures (only the working memory task is relevant for the current experiment). Suitable participants advanced to the subsequent 2 EEG testing sessions, during which participants received an oral capsule of immediate-release 20 mg methylphenidate (MPH) or an identically overcoated placebo. MPH is one of the most widely prescribed drugs for the treatment of attention-deficit/hyperactivity disorder (ADHD) (51). It works by blocking the reuptake of dopamine and noradrenaline, and thus increases extracellular catecholamine levels (41). Previous studies have demonstrated that MPH administration effectively elevates plasma catecholamine levels. Additionally, it causes cardiac effects, which are mediated via peripheral β1 adrenoceptors, indicating that methylphenidate has a systemic impact on cardiovascular function (52, 53). These well-established peripheral and central nervous system effects can be used to help confirm whether the drug reached both the systemic circulation and its target organs, ensuring the desired pharmacological outcomes. The dose of 20 mg MPH has been shown to be safe, as well as sufficient to influence cognitive functions such as cognitive control and language processing (9, 35, 42). Half of the participants received MPH in EEG session 1 and placebo in EEG session 2, while the other half received the drug/placebo administration in reversed order. Both participant and responsible researcher were unaware of whether the participant received MPH or placebo at the time of testing. The two EEG sessions were separated by at least 1 wk (mean = 18.5 d, SD = 39.5; because of the special testing rules during the COVID-19 period, the interval between the two sessions was longer, and more variable than normal) to ensure drug washout.

In each EEG session, after the capsule administration participants were instructed to complete a series of tasks, including a sentence comprehension task, a spontaneous eye blink rate recording, several questionnaires (reported below), and some other cognitive performance measures (e.g., Go/No-go task; these data will be reported in a separate paper). The starting time of the first task, the sentence comprehension task, was set at approximately 90 min (mean = 90 min, SD = 3 min) after capsule administration. This was timed to coincide with the expected peak concentration of MPH in the plasma, which on average occurs around 1 to 1.5 h postadministration (42, 54). During each EEG session, the participants’ heart rate (HR), systolic blood pressure (SBP), and diastolic blood pressure (DBP) were monitored three times: at the beginning of the session (about 5 min after arriving at the lab), before starting the sentence comprehension task (about 1.5 h after capsule administration), and at the end of the session (about 4 h after capsule administration). In addition, the participants’ subjective feeling and drug-related physical complaints were assessed with a digital version of the Bond-Lader visual analogue scale (BL-VAS) (55) and a physical symptoms questionnaire (53), respectively. In the VAS, the participants were asked to provide self-evaluation on 16 questions by adjusting a sliding bar on a continuum between two opposing states, e.g., “alert-drowsy,” “calm-excited,” and “strong-weak.” The individual responses from the 16 questions were combined to make three affective dimensions of alertness, contentment, and calmness. BL-VAS has previously been successfully used to quantify participants’ subjective feelings and has been extensively used to delineate drug effects in psychopharmacological studies. Similarly, in the physical symptoms questionnaire (53), the participants were asked to do self-report on 10 questions, e.g., headache, dry mouth, etc. These two tasks were administered at both the beginning and the end of each EEG session.

### 3.3. Procedure.

#### 3.3.1. Sentence comprehension task.

The sentence materials consisted of 240 sets of Dutch garden-path sentences, which were selected and modified from a set of stimuli used in a previous study (23). Each set of critical sentences comprised two versions (ambiguous vs. unambiguous conditions). All sentences followed the structure: noun phrase (NP) 1 + verb phrase (VP) 1 + NP2 + “and” + NP3 + VP2 +…, with identical lexical items up to the VP2 within each set (see examples in Table 1). The average length of the critical sentences was 12.8 words (*SD* = 1.5). The critical word was always the 9th word and it was never in the sentence-final position. Corresponding filler sentences were created for each of the 240 critical sentences by replacing the VP2 with a subordinate conjunction (e.g., because, that, once) thus removing any structural ambiguity (e.g., “The fan attacked the drummer and the guitarist because the music was too loud.”). An additional 36 filler sentences with a different sentence structure (e.g., “The barista served the singer and the drummer and the guitarist, after which he cleaned the bar.”) were also included to further counteract participants developing any particular processing strategy. Moreover, 200 grammatical and meaningful sentences designed for a different experimental purpose were included as fillers (e.g., “The actor made jokes all evening.”).

To avoid repetition of sentence content within participants, each participant read only one variant from each critical sentence set, resulting in a total of 160 critical sentences (80 each from the Ambiguous and Unambiguous conditions), and 316 filler sentences in each reading list. Three lists were created with two pseudorandomized orders for each one, making a total of six experimental lists. Participants were randomly assigned to one list and read the first half during EEG session 1 and the second half during EEG session 2.

During the experiment, participants were instructed to silently read sentences presented on the computer screen in front of them, and then answer a question following each sentence. The sentence was presented in a word-by-word fashion. Each sentence started with a fixation cross in the center of the screen for 1,000 to 1,500 ms. Then each word was presented for 200 ms followed by a blank screen for 600 ms. The fifth word in the Unambiguous condition was presented with a comma, while the final word of all sentences was presented with a period. A comprehension question (e.g., “Did the fan pack the instruments?”) was presented for up to 5 s or until participants pressed a button to indicate their answer. The same comprehension question was presented after each version of a sentence (Ambiguous/Unambiguous/filler) from each set. Participants were asked to press the left or the right button with their right hand on a button box to provide a “Yes” or “No” answer. Half of the questions would be correctly answered with No and the other half with Yes. The response mapping was counterbalanced across participants. Twelve practice sentences were presented directly before the sentence task in each EEG session to help participants get familiar with the task. There was a 1.5 min break after every 45 sentences, and the entire sentence comprehension experiment lasted about 60 to 70 min.

#### 3.3.2. Individual differences in catecholamine drug effects: WM tasks.

Given extensive prior evidence suggesting that catecholamine drug effects depend on individual differences in baseline working memory capacity (3, 33, 34, 37, 56, 57), we assessed participants’ WM capacity. The relationship between WM capacity and catecholamine levels remains a topic of debate. Early PET studies suggested that WM capacity might serve as a proxy of individual striatal DA levels, with higher WM capacity associated with greater striatal DA synthesis capacity and more efficient dopaminergic transmission (58). However, these results were not replicated in a recent PET study with a large sample of 100 participants (52). Thus, no final conclusion has been reached regarding the link between WM capacity and dopaminergic drug effects.

During the intake session, participants’ WM capacity was assessed with the automatic reading span and operation span tasks (59). In both tasks, participants had to memorize a series of letters while simultaneously performing a distractor task, which involved reading a sentence in the reading span task or solving a simple math operation in the operation span task. Participants’ WM performance was evaluated as the total number of correctly recalled letters in each task. Working memory capacity was indexed by a composite score by combining the standardized z-scores from both the reading span and operation span tasks. This composite WM score of WM capacity was included as a fixed effects predictor of interest in the exploratory analyses of our behavioral and EEG data.

#### 3.3.3. Individual differences in catecholamine drug effects: Spontaneous eye blink rate.

Spontaneous eye blink rate has been proposed as a noninvasive biomarker of DA activity, although the exact association and its physiological nature remain unclear. Some studies suggest positive correlations (60), whereas others report negative correlations (61), and some find no correlation at all (52, 62). These discrepancies may reflect differences in drugs and species used in the different studies. In this study, we tested the hypothesis that catecholamine drug effects vary with spontaneous eye blink rate by comparing blink rate following placebo and MPH administration.

During each EEG session, EEG data were recorded during a resting state task prior to the sentence comprehension experiment. Participants were instructed to fixate a central fixation cross and then either maintained fixation with their eyes open for 5 min (Eyes Open data), or closed their eyes for 5 min until the researcher instructed them to open their eyes again (Eyes Closed data). The Eyes Open data were used to calculate participants’ sEBR and to test whether these differed between the session where they had been administered the drug compared with when they had been administered the placebo.

### 3.4. EEG Data Acquisition.

Participants were seated in the EEG booth wearing a customized EEG cap (actiCAP), facing a computer monitor at a distance of approximately 70 cm from the screen. EEG signals were recorded from 26 Ag/AgCl active electrodes mounted in the cap according to the international 10 to 20 system (Fp1/2, Fz, F3/4, F7/8, FCz, FC1/2, FC5/6, Cz, C3/4, CP1/2, CP5/6, Pz, P3/4, P7/8, and O1/2). Bipolar horizontal EOG was derived from two additional electrodes placed at the left and right outer canthi of the participant, and bipolar vertical EOG was derived from electrode Fp1 in the EEG cap and an additional electrode placed under the participant’s left eye. During the recording, all electrodes were referenced to an online reference electrode placed on participants’ left mastoid, and the ground electrode was placed in the center of their forehead. One additional electrode was placed on participants’ right mastoid to be used for offline rereferencing. Impedances were kept below 25 kΩ for all electrodes. The EEG and EOG signals were digitized online with a 500-Hz sampling frequency and amplified through a BrainVision DC amplifier with a 200 Hz low-pass filter and a 0.016 Hz high-pass filter.

### 3.5. Data Analysis.

#### 3.5.1. Sentence comprehension task: Behavioral data.

For the sentence comprehension experiment, the reaction times (RTs) and the accuracy data were analyzed by Bayesian linear mixed-effects regression analyses using the brms package (63) in R (R version 4.3.1). This Bayesian model was specifically chosen to ensure stable estimation of higher-order interactions given our final sample size (N = 30), as it is more robust to smaller cohorts and avoids the convergence issues (e.g., singular fits) often encountered in frequentist maximum likelihood estimation (64, 65).The dependent variables were RTs and accuracy data in each model. The accuracy data were modeled employing logistic regression with a logit link function to predict binary variables, while the RT data were modeled employing a standard Gaussian estimation approach. The fixed effects predictors included in each model were: Drug (Placebo vs. Methylphenidate), Ambiguity (Ambiguous vs. Unambiguous), Drug Order (the participants assigned to Order 1 took methylphenidate in session 1, n = 14; while those assigned to Order 2 took methylphenidate in session 2, n = 16), as well as the interactions among the three predictors. The Drug Order predictor was included to examine any potential differential influence of MPH on garden-path sentence processing depending on whether the drug was administered in the first or the second EEG session. Previous studies have shown that repeated practice or cognitive training improves individuals’ ambiguity resolution ability during language comprehension (66), and such effects may reasonably be expected to interact with the order in which the drug/placebo was administered. The random effects structure included by-participant and by-item intercepts and slopes for Drug, Ambiguity, and their interaction. Drug, Ambiguity, and Drug Order were contrast coded as −1 (Placebo/Ambiguous/Drug Order1) and 1 (Methylphenidate/Unambiguous/Drug Order2).

Regularizing, weakly informative priors were used as follows: all fixed effects parameters, including the intercept were drawn from a normal distribution with mean of 0 and SD of 1; random effects and residual error parameters were drawn from a truncated (positive only) normal distribution with mean of 0 and SD of 5; correlation parameters were based on LKJ-correlation priors, with numerical parameter of 2 – LKJ (2). The models assumed a linear Gaussian response distribution and empirical posterior estimation for parameters was carried out via the No-U-Turn sampler (v2.320.6; 4 separate Markov chains; 10,000 sampling iterations per chain, of which 2,000 samples were reserved for burn-in) (67). Model convergence was assessed by inspecting both R statistic values (*Ȓ*) to ensure that they were within an acceptable range, and trace plots for all parameters to ensure that Markov chains were well mixed. We treat an effect as providing reliable evidence when the 95% CrI of its posterior estimate excludes zero, and as providing suggestive evidence when the 90% CrI excludes zero. These labels are intended as descriptive shorthand summarizing the precision of parameter estimation. Following current recommendations for Bayesian inference (68), we report the full posterior estimate (mean and CrI) for every effect of interest in *SI Appendix*, *Supplementary Materials*, so that the magnitude and uncertainty of each finding can be evaluated alongside the verbal labels applied.

#### 3.5.2. Cardiovascular measurements.

For the cardiovascular responses and BL-VAS ratings, the results were examined with a 2 Drug (Placebo vs. MPH) ×2 Time (Before vs. After) repeated measures ANOVA with Drug Order (MPH-PLA vs. PLA-MPH) as a between-participant factor using R software. The Before level of the Time factor refers to measurements made at the very beginning of each EEG session, preceding the administration of the drug/placebo; while the After level refers to measurements made at the end of each EEG session, about 4 h after the drug/placebo administration. For the spontaneous eye blink rate data, the analysis followed a similar procedure, with the exclusion of the Time factor, since spontaneous eye blink rate was only measured once, after drug/placebo administration.

#### 3.5.3. EEG data: Sentence comprehension task.

EEG data from each of the two sessions were preprocessed separately using the FieldTrip toolbox (69) running in a MATLAB environment. Data were high-pass filtered at 0.3 Hz and a band-stop filter was applied at 50, 100, and 150 Hz to minimize any influence of slow drift and power line noise respectively. Epochs were then created from 1,200 ms before to 1,300 ms after the onset of the critical word (e.g., “pakte” in the example sentences in Table 1) of each sentence, and the data were rereferenced to the mean amplitude of the left and right mastoid electrodes (average mastoid reference). In a first round of artifact rejection, trials with severe artifacts unrelated to eye blinks or eye movements were identified via visual inspection, and together with any bad channels these were removed from the data. Next, independent component analysis (ICA) was carried out using EEGLAB’s “runica” implementation in FieldTrip with default settings, and components capturing eye blinks, eye movements, and cardiac artifacts were removed from the data (70). Any remaining artifact trials were removed via visual inspection, and bad channels were recovered from a combination of neighboring electrodes. Finally, epochs were shortened to −200 to 1,000 ms relative to critical word onset, and a 30 Hz low-pass filter was applied to the data. For the purposes of creating ERP figures, a baseline correction was applied using a baseline period of −200 to 0 ms relative to critical word onset. For statistical analyses no baseline correction was applied, but instead the mean amplitude in the baseline period was extracted on a single-trial basis and included as a predictor of no interest in mixed effects regression modeling analyses of the ERP data (71).

Two time windows were selected based on previous studies (9, 22). First, the 550 to 900 ms time window was of most interest. It was selected to capture the P600 effect, which has been consistently observed in locally syntactically ambiguous sentences at the point of disambiguation (22, 23). Second, the 250 to 500 ms time window was selected to capture the N400 effect, a well-established ERP effect related to semantic processing (10). The amplitude of the N400 response is sensitive to the difficulty of semantic processing, with larger amplitudes indicating greater processing effort. Since Tan and Hagoort’s study (9) showed that MPH can have different effects on early and late N400 time windows, we selected an early N400 time window of 250 to 350 ms, and a later N400 time window of 350 to 500 ms. Mean EEG amplitudes within each of these predefined time windows were extracted on a single-trial basis, and analyzed by Bayesian linear mixed-effects regression analyses using the *brms* package. The models mirrored those for the RT data as described above but included an additional fixed effects predictor for the Region of Interest (ROI) over the scalp where the electrodes were positioned. For lateral electrodes the following four ROIs were chosen: Left-Anterior (F3, F7, FC1, FC5), Right-Anterior (F4, F8, FC2, FC6), Left-Posterior (CP1, CP5, P3, P7), and Right-Posterior (CP2, CP6, P4, P8). ROI values were computed as the mean amplitude over the four electrodes within each region. A contrast matrix was created to compare anterior versus posterior electrodes (contrast codes: Left-Anterior 0.5, Right-Anterior 0.5, Left-Posterior −0.5, Right-Posterior −0.5), and left vs. right electrodes (contrast codes: Left-Anterior 0.5, Right-Anterior −0.5, Left-Posterior 0.5, Right-Posterior −0.5). A second set of analyses including ROI were carried out for the midline, comparing the mean amplitude over the Anterior-Middle region (Fz, FCz; coded as −1) with that over the Posterior-Middle region (Cz, Pz; coded as 1).

Additionally, we conducted an exploratory analysis by rerunning the statistical models for the critical word, including WM as a predictor. These models mirrored those for the behavioral and EEG data as described above, but included the main effect of WM as well as its interactions with other fixed effects predictors. WM was also incorporated into the by-participant adjustments for the random slopes. To avoid overfitting, we simplified the models by removing the Drug Order predictor, which was of least interest and had limited impact in the analyses without the WM predictor. Although the inverted-U is the canonical functional form for catecholaminergic modulation of cognition (3), we estimated the baseline dependency of MPH effects with a linear interaction model. This choice was driven by two considerations. First, our sample size (N = 30) constrains the complexity of the functional forms that can be reliably estimated. Second, the WM capacity range in our participants was narrower than the range typically required to detect curvature. To limit the risk of overfitting given these constraints, we used weakly informative priors throughout our models, which keep posterior estimates from being driven by extreme values in the data (65). Larger cohorts spanning a broader WM range—particularly those including clinical or older participants—will be needed to fit explicit nonlinear models designed to investigate this kind of relationship.

Last, we investigated whether a CPS was detectable in a 400 to 800 ms time window time-locked to the onset of the second noun (“drummer” in the example materials in Table 1), where a comma is either present and clearly marks a prosodic phrase boundary (Unambiguous condition) or is absent (Ambiguous condition) and the appropriate phrase boundary is not explicitly marked in the visual input during reading. The presence vs. absence of prosodic phrase boundaries has been associated with the CPS component (22, 40, 72), which has also been observed in reading tasks where phrase boundaries are visually marked with a comma as in our experimental stimuli (22, 23, 40). These analyses aimed to assess whether MPH influences readers’ processing of prosodic phrase boundaries, and more importantly, whether its effects target general arousal (i.e., affecting the processing of all words within the sentences) or instead are specific to syntactic unification demands resulting from a need for ambiguity resolution (i.e., affecting processing specifically at the critical word). All preprocessing details apart from the chosen time windows were the same as for the analyses at the critical word, and Bayesian linear mixed-effects regression analyses were again used to assess statistical significance in the same way as was done for the critical word.

#### 3.5.4. sEBR.

Eyes Open data from each of the two EEG sessions were processed separately, and precisely the same preprocessing steps as in the sentence comprehension task were applied to these data. These data were epoched from the onset of the fixation period to the end of the fixation task. The first and last 5,000 ms of the data were discarded to avoid artifacts near the beginning and end of the recordings, and the remaining data were segmented into 1,000 ms epochs with 500 ms overlap. In order to detect eye blinks in a principled manner, we extracted the time course of the independent component that was identified as capturing eye blinks in each participant and session, applied a high-pass filter at 1 Hz, a low-pass filter at 10 Hz, removed the DC offset, and applied a median filter (temporal smoothing) of order 9. A threshold was set such that any epochs in which the amplitude of the waveform of this component fell more than 1.5 SD above the mean of the absolute amplitude across all epochs or more than 1.5 SD below the negative mean of the absolute amplitude across all epochs were marked as eye blinks. Since each epoch was 1,000 ms, sEBR was calculated by counting the total number of blinks that were detected in all epochs, and dividing by the number of epochs, resulting in a sEBR expressed in blinks per second. A 2(Drug) × 2(Drug Order) repeated-measures ANOVA was conducted using R software to analyze differences between methylphenidate and placebo sessions.

## Supplementary Material

Appendix 01 (PDF)

## Data Availability

Anonymized ERP data have been deposited in the Radboud Data Repository (73).
