# Supplementary material for: Neuropharmacology of language: ERP evidence for catecholaminergic modulation of syntactic processing
Source: Proc Natl Acad Sci U S A. 2026 Oct 22;123(43):e2606806123. doi: 10.1073/pnas.2606806123 (PMC13715833; doi:10.1073/pnas.2606806123)

Supplementary Materials

Supplementary Table A. MPH effect on ambiguity resolution: the model outputs for the Bayesian linear mixed-effects regression analyses time-locked to the critical word (e.g., "*packed*")

Time	Predictor	Lateral electrodes			Midline electrodes		
		Posterior mean	95% CrI	SD	Posterior mean	95% CrI	SD
N400 early (250 – 350 ms)							
	Drug	.01	[-.04, .06]	.02	.01	[-.05, .07]	.03
	Drug×Amb *	-.03	[-.06, .01]	.02	-.03	[-.07, .01]	.02
	Drug×Ord	-.03	[-.07, .02]	.02	-.02	[-.08, .04]	.03
	Drug×Amb×Hemi	-.01	[-.04, .02]	.02			
	Drug×Amb×Ant	.01	[-.02, .04]	.02	.00	[-.02, .01]	.01
	Drug×Amb×Ord	-.02	[-.05, .01]	.02	-.02	[-.06, .02]	.02
	Drug×Hemi×Ord	.02	[-.01, .05]	.02			
	Drug×Ant×Ord	-.01	[-.04, .03]	.02	-.01	[-.03, .00]	.01
	Drug×Amb×Hemi×Ord	.01	[-.02, .04]	.02			
	Drug×Amb×Ant×Ord	.02	[-.01, .05]	.02	-.01	[-.02, .01]	.01
	Amb	.01	[-.03, .04]	.02	.00	[-.04, .04]	.02
	Amb × Hemi	.01	[-.02, .04]	.02			
	Amb × Ant	.01	[-.02, .04]	.02	.00	[-.02, .01]	.01
	Amb × Order	-.01	[-.05, .03]	.02	-.02	[-.06, .03]	.02
	Amb × Hemi × Order	.01	[-.03, .04]	.02			
	Amb × Ant × Order	-.01	[-.04, .03]	.02	.01	[-.01, .02]	.01
N400 late (350 – 500 ms)							
	Drug	.00	[-.05, .05]	.03	.00	[-.05, .06]	.03
	Drug×Amb	-.01	[-.04, .03]	.02	.00	[-.04, .04]	.02
	Drug×Ord	-.02	[-.07, .03]	.03	-.03	[-.08, .03]	.03
	Drug×Amb×Hemi	.00	[-.04, .03]	.02			
	Drug×Amb×Ant	.00	[-.03, .03]	.02	.00	[-.01, .02]	.01
	Drug×Amb×Ord	-.02	[-.06, .01]	.02	-.02	[-.06, .02]	.02
	Drug×Hemi×Ord	.02	[-.02, .05]	.02			
	Drug×Ant×Ord	.00	[-.04, .03]	.02	-.01	[-.02, .01]	.01
	Drug×Amb×Hemi×Ord	.00	[-.04, .03]	.02			
	Drug×Amb×Ant×Ord ·	.02	[-.01, .06]	.02	.00	[-.02, .01]	.01
	Amb	.01	[-.02, .05]	.02	.00	[-.04, .04]	.02
	Amb × Hemi	.02	[-.02, .05]	.02			
	Amb × Ant	-.01	[-.04, .03]	.02	.00	[-.01, .02]	.01
	Amb × Order	.00	[-.04, .04]	.02	.00	[-.05, .04]	.02
	Amb × Hemi × Order	.02	[-.02, .05]	.02			
	Amb × Ant × Order	-.01	[-.05, .02]	.02	.01	[-.01, .03]	.01
P600 (550 – 900 ms)							
	Drug	.00	[-.02, .02]	.01	.00	[-.02, .02]	.01
	Drug×Amb	.01	[-.01, .03]	.01	.01	[.00, .03]	.01
	Drug×Ord	.00	[-.02, .02]	.01	-.01	[-.03, .02]	.01
	Drug×Amb×Hemi	.00	[-.01, .01]	.01			
	Drug×Amb×Ant *	.01	[.00, .02]	.01	.01	[.00, .01]	.00
	Drug×Amb×Ord	.00	[-.01, .02]	.01	.01	[-.01, .02]	.01
	Drug×Hemi×Ord	.00	[-.01, .01]	.01			
	Drug×Ant×Ord	.00	[-.01, .01]	.01	.00	[-.01, .01]	.00
	Drug×Amb×Hemi×Ord	.00	[-.01, .02]	.01			
	Drug×Amb×Ant×Ord	.00	[-.01, .02]	.01	.00	[.00, .01]	.00

Amb	-.01	[-.03, .01]	.01	-.01	[-.03, .01]	.01
Amb × Hemi	.00	[-.01, .01]	.01			
Amb × Ant *	.01	[.00, .02]	.01	.01	[.00, .01]	.00
Amb × Order	.00	[-.01, .02]	.01	.00	[-.02, .02]	.01
Amb × Hemi × Order	.00	[-.01, .02]	.01			
Amb × Ant × Order	-.01	[-.02, .01]	.01	.00	[-.01, .00]	.00
Exploratory analysis N150-200 (150 – 200 ms)						
Drug ·	.03	[-.02,.07]	.02	.03	[-.01,.07]	.02
Drug×Amb *	-.03	[-.06,.00]	.02	-.02	[-.06,.02]	.02
Drug×Ord	-.03	[-.08,.01]	.02	-.03	[-.08,.01]	.02
Drug×Amb×Hemi	.00	[-.03,.03]	.01			
Drug×Amb×Ant	.00	[-.03,.02]	.01	.00	[-.02,.01]	.01
Drug×Amb×Ord	-.01	[-.04,.02]	.02	-.01	[-.05,.03]	.02
Drug×Hemi×Ord	-.01	[-.04,.02]	.01			
Drug×Ant×Ord	.00	[-.03,.03]	.01	-.01	[-.02,.01]	.01
Drug×Amb×Hemi×Ord	.00	[-.02,.03]	.01			
Drug×Amb×Ant×Ord	.01	[-.02,.04]	.01	.00	[-.01,.01]	.01
Amb	.01	[-.03,.04]	.02	.00	[-.04,.04]	.02
Amb × Hemi	.01	[-.02,.04]	.01			
Amb × Ant	.02	[-.01,.04]	.01	-.01	[-.02,.00]	.01
Amb × Order	.02	[-.02,.05]	.02	.02	[-.02,.06]	.02
Amb × Hemi × Order	.00	[-.03,.03]	.01			
Amb × Ant × Order	-.02	[-.04,.01]	.01	.01	[-.01,.02]	.01

Note.

1) "Amb" represents the predictor for Ambiguity; "Ant" represents the predictor for Anteriority (Anterior vs. Posterior electrodes); "Hemi" represents the predictor for Hemisphere (Left vs. Right electrodes); “Ord” represents the predictor for Drug Order (MPH-PLA vs. PLA-MPH).

2) An asterisk (*) indicates results where zero falls outside the 95% CrI of the posterior estimates. A dot (·) indicates results where zero falls outside the 90% CrI of the posterior estimates. These results are presented in bold fonts.

3) In the late N400 time window, there was some evidence for a four-way interaction among Drug, Ambiguity, Anteriority, and Drug Order at lateral electrodes (estimated posterior distribution >0 = 91.2%), which did not meet our criteria for statistical significance. Planned analyses indicated that the Drug × Ambiguity × Anteriority interaction showed a tendency towards opposite directions depending on whether the participants received MPH in session 1, while none of the further analyses reached statistical significance.

Supplementary Table B. Posterior estimates of fixed effects incorporating of WM capacity on ERP mean amplitude time-locked to the critical word (e.g., "*packed*") in Bayesian regression models

Time	Predictor	Lateral electrodes			Midline electrodes		
		Posterior mean	95% Crl	SD	Posterior mean	95% Crl	SD
N400 early (250 – 350 ms)							
	WM	-.06	[-0.17, 0.03]	.05	-.09	[-.21, .02]	.06
	Drug×WM	.01	[-0.02, 0.05]	.02	.01	[-.02, .05]	.02
	Cond×WM	.00	[-0.03, 0.02]	.01	-.01	[-.04, .02]	.01
	Drug×Cond×WM	.01	[-0.01, 0.03]	.01	.01	[-.02, .03]	.01
	Drug×Hemi×WM	.00	[-0.02, 0.02]	.01			
	Drug×Ant×WM	-.00	[-0.02, 0.02]	.01	.00	[-.01, .01]	.00
	Cond×Hemi×WM	.00	[-0.02, 0.02]	.01			
	Cond×Ant×WM	.01	[-0.01, 0.03]	.01	.00	[-.01, .01]	.00
	Drug×Cond×Hemi×WM	.00	[-0.02, 0.02]	.01			
	Drug×Cond×Ant×WM	.01	[-0.01, 0.03]	.01	.00	[-.01, .01]	.00
N400 late (350 – 500 ms)							
	WM	.01	[-0.04, 0.07]	.03	.00	[-.07, .06]	.03
	Drug×WM	-.01	[-0.04, 0.02]	.02	-.01	[-.04, .03]	.02
	Cond×WM	.01	[-0.02, 0.03]	.01	.01	[-.02, .03]	.01
	Drug×Cond×WM	.00	[-0.02, 0.02]	.01	.00	[-.03, .02]	.01
	Drug×Hemi×WM	.01	[-0.01, 0.03]	.01			
	Drug×Ant×WM	.00	[-0.02, 0.02]	.01	.00	[-.01, .01]	.00
	Cond×Hemi×WM	.00	[-0.02, 0.02]	.01			
	Cond×Ant×WM	.00	[-0.02, 0.02]	.01	.00	[-.01, .01]	.00
	Drug×Cond×Hemi×WM	.00	[-0.01, 0.02]	.01			
	Drug×Cond×Ant×WM	-.01	[-0.01, 0.03]	.01	.00	[-.01, .01]	.00
P600 (550 – 900 ms)							
	WM	.00	[-.02, .03]	.05	.00	[-.02, .03]	.01
	Drug×WM	.00	[-.01, .01]	.02	.00	[-.02, .01]	.01
	Cond×WM	.00	[-.01, .01]	.01	.00	[-.01, .01]	.00
	Drug×Cond×WM	.00	[-.01, .01]	.01	.00	[-.01, .01]	.01
	Drug×Hemi×WM	.00	[-.01, .01]	.01			
	Drug×Ant×WM	.00	[-.01, .01]	.01	.00	[.00, .00]	.00
	Cond×Hemi×WM	.00	[-.01, .01]	.01			
	Cond×Ant×WM *	.01	[.00, .02]	.01	.00	[-.01, .00]	.00
	Drug×Cond×Hemi×WM	.00	[-.01, .00]	.01			
	Drug×Cond×Ant×WM *	-.01	[-.01, .00]	.01	.00	[.00, .01]	.00

Note: All the predictors that include WM are shown in the table. "Amb" represents the predictor for Ambiguity; "Ant" represents the predictor for Anteriority (Anterior vs. Posterior electrodes); "Hemi" represents the predictor for Hemisphere (Left vs. Right electrodes). An asterisk (*) denotes results where the posterior distribution indicated a greater than 95% probability of non-zero values. These results are presented in bold fonts.

Supplementary Table C. Posterior estimates of fixed effects of Drug manipulation on ERP mean amplitude time-locked to the second noun (e.g., "*drummer*") in Bayesian regression models

Time	Predictor	Lateral electrodes			Midline electrodes		
		Posterior mean	95% Crl	SD	Posterior mean	95% Crl	SD
CPS (400 – 800 ms)							
	Drug	0.01	[-0.02, 0.05]	0.02	0.00	[-0.04, 0.04]	0.02
	Drug×Bound	0.00	[-0.03, 0.03]	0.01	0.01		0.02
	Drug Ord	-0.03	[-0.07, .00]	0.02	-0.03	[-0.07, 0.01]	0.02
	Drug×Bound×Hemi	0.00	[-0.02, 0.03]	0.01			0.01
	Drug×Bound×Ant	0.01	[-0.02, 0.04]	0.01	0.00	[-0.02, 0.01]	0.02
	Drug×Bound×Ord	-0.01	[-0.04, 0.02]	0.02	-0.01	[-0.05, 0.02]	0.01
	Drug×Hemi×Ord	0.00	[-0.03, 0.03]	0.01			0.01
	Drug×Ant×Ord	-0.01	[-0.04, 0.02]	0.01	0.00	[-0.01, 0.02]	0.01
	Drug×Bound×Hemi×Ord	0.02	[.00, 0.05]	0.01			0.01
	Drug×Bound×Ant×Ord	-0.02	[-0.04, 0.01]	0.01	0.01	[-0.01, 0.02]	0.01
	Bound	-0.01	[-0.03, 0.02]	0.01	-0.01	[-0.03, 0.02]	0.01
	Bound × Hemi	-0.01	[-0.03, 0.02]	0.01			0.02
	Bound × Ant	0.02	[-0.01, 0.05]	0.01	-0.01	[-0.02, 0.01]	0.02
	Bound × Order	-0.01	[-0.04, 0.01]	0.01	-0.01	[-0.03, 0.02]	0.02
	Bound × Hemi × Order	0.01	[-0.02, 0.04]	0.01			0.01
	Bound × Ant × Order	0.00	[-0.02, 0.03]	0.01	0.00	[-0.02, 0.01]	0.02

Note. a) In the table, Drug-related effects are presented in black ink to emphasize their primary focus in the analysis, while Ambiguity-only effects are presented in gray ink. To ensure the primary variables of interest are clearly highlighted, other effects that do not include either Drug or Ambiguity predictors are not presented here, as they are not the focus of the current study. "Bound" represents the predictor for Boundary effect; "Ant" represents the predictor for Anteriority effects (Anterior vs. Posterior electrodes); "Hemi" represents the predictor for Hemisphere effects (Left vs. Right electrodes); "Ord" represents the predictor for Order effects (MPH-PLA vs. PLA-MPH). An asterisk (*) indicates results where zero falls outside the 95% CrI of the posterior estimates. These results are presented in bold fonts.

Supplementary Table D. Sentence behavioral results: the full model outputs for the Bayesian linear mixed-effects regression analyses

DV	Predictor	Posterior mean	95% Crl	SD
Accuracy	Drug	.11	[-.07, .30]	.09
	Amb	.05	[-.05, .14]	.05
	Ord	.05	[-.25, .35]	.15
	**Drug × Amb** ·	**-.09**	**[-.22, .03]**	**.06**
	Drug × Ord	-.07	[-.28, .15]	.11
	Amb × Ord	.03	[-.06, .13]	.05
	Drug × Amb× Ord	-.03	[-.15, .08]	.06
RT	**Drug** *	**-.08**	**[-.13,-.02]**	**.03**
	**Amb** ·	**-.02**	**[-.05, .01]**	**.01**
	Ord	.05	[-.12, .22]	.09
	Drug × Amb	-.01	[-.03, .02]	.01
	Drug × Ord	.01	[-.07, .08]	.04
	**Amb × Ord** ·	**.02**	**[-.01, .05]**	**.01**
	Drug × Amb× Ord	0	[-.03, .02]	.01

Note. For the RT data, there was some inconclusive evidence for an interaction between Ambiguity and Drug Order with 93.6% of posterior estimates falling above zero. Planned analyses showed that the Ambiguity effect was statistically significant for participants who received MPH in session 1 (mean = -0.04, 95% CrI [-0.10, 0.01], posterior estimates > 0 = 96.1%), but not for participants who received MPH in session 2 (mean = -0.01, 95% CrI [-0.09, 0.07]). Nonetheless, given that zero falls within the 95% CrI of the posterior estimates for the Ambiguity × Drug Order interaction, it is necessary to recognize that the findings from these posthoc analyses should not be considered conclusive.

Supplementary Table E. Cardiovascular and general modulation effect of MPH

			Placebo	MPH	Differences
Diastolic blood pressure		Before	72.5 (8.1)	71.8 (8.0)	t (29) = .71, p = .482
		After	72.7 (10.1)	73.9 (8.5)	t (29) = -1.28, p = .211
Systolic blood pressure		Before	121.3 (10.6)	118.2 (12.2)	t (29) = 2.73, p = .011*
		After	115.1 (10.1)	119.6 (9.6)	t (29) = -4.32, p < .001**
Heart rate		Before	76.3 (14.8)	73.8 (13.5)	t (29) = 1.14, p =.264
		After	62.2 (11.5)	72.4 (12.6)	t (29) = -6.40, p < .001**
BL-VAS	Alertness	Before	2.8 (1.2)	2.6 (1.1)	t (29) = .81, p = .423
		After	4.3 (1.4)	3.1 (1.3)	t (29) = 6.51, p < .001**
	Calmness	Before	3.4 (1.5)	3.1 (1.5)	t (29) = 1.01, p = .322
		After	2.2 (1.4)	3.6 (1.8)	t (29) = -4.73, p < .001**
	Contentment	Before	2.6 (1.1)	2.4 (1.0)	t (29) = 1.04, p = .309
		After	2.7 (1.3)	2.5 (1.1)	t (29) = 1.25, p = .221
Blink rate (count/s)			0.78 (0.48)	0.77 (0.51)	F (1, 28) < 1

Note. a. "Before" indicates measurements taken prior to capsule administration, while "After" indicates measurements taken approximately 4 hours after capsule administration. Values in parentheses represent the standard deviation from the mean.

b. Lower scores on the BL-VAS questionnaire indicate higher levels of alertness, contentment, and calmness. Each dimension score was derived from the average responses on the questionnaire.

c. The last column displays results of paired-sample t-tests comparing the placebo and MPH conditions for each dependent variable. * indicates $p < .05$; ** indicates $p < .001$.

Cardiovascular responses. There were statistically significant interactions between Drug (MPH vs. Placebo) and Time (before vs. after) for heart rate, F (1, 28) = 33.13, $p < .001$; and for systolic blood pressure, F (1, 28) = 22.63, $p < .001$. As shown in Table E, planned post-hoc analyses revealed that before drug administration, there was no statistically significant difference between the MPH and the placebo conditions for the heart rate. Participant’s systolic blood pressure was even higher in the placebo condition than the MPH condition. However, after drug administration, both participants’ heart rate and systolic blood pressure were higher in the MPH condition than the placebo condition. The interaction between MPH and Time was not significant for diastolic blood pressure, F (1, 28) = 2.39, $p = .133$. The Drug Order effect was neither statistically significant, nor did it interact with the MPH or Time predictors. These findings align with previous studies involving MPH manipulation, which have shown that MPH administration leads to increased cardiovascular responses (Tan & Hagoort, 2020).

BL-VAS questionnaire (Bond & Lader, 1974). Participants’ self-ratings on the BL-VAS were assessed across three dimensions: alertness, calmness and contentment. There were statistically significant interactions between Drug and Time for the alertness ratings, F (1, 28) =

16.18, p <.001, and for the calmness ratings, F (1, 28) = 15.81, p < .001. As shown in Table E, post-hoc analyses revealed that before drug administration, there were no statistically significant differences between the MPH and the placebo conditions for either the alertness ratings or the calmness ratings. However, after drug administration, participants showed higher alertness and lower calmness in the MPH than the placebo condition. There was no effect related to the Drug order factor. In addition, there was a three-way interaction between Drug, Time, and Drug Order for the contentment ratings, F (1, 28) = 5.76, p = .023. Further analyses showed that for participants who received MPH in session 1, there was a significant interaction between Drug and Time, F (1, 28) = 5.36, p = .038. These participants showed matched contentment levels between the MPH and placebo conditions in the Before measurements (mean rating: placebo = 2.6 vs. MPH = 2.7; t (29) = -0.97, p = 0.35), but reported higher contentment in the MPH than placebo condition in the After measurements (mean rating: placebo = 3.1 vs. MPH = 2.7; t (29) = 2.56, p = .024). Nonetheless, no such effect was observed among participants who received MPH in session 2.

Spontaneous eye blink rate (sEBR). As shown in Table E, there was no statistically significant change in participants' sEBR following MPH administration compared with placebo, F (1, 28) < 1. Moreover, the Drug Order effect was neither statistically significant, nor did it exhibit any statistically significant interactions with any other factors (F s < 1).

Together, through cardiovascular assessments and self-report questionnaires, we demonstrated the impact of our MPH manipulation. Following MPH administration, participants exhibited increased heart rate and systolic blood pressure compared to the placebo condition. They also subjectively reported heightened alertness and reduced calmness. Additionally, participants who received MPH in session 1 also reported higher contentment in the MPH condition post-administration compared with the placebo condition. These results show that drug administration resulted in adequate systemic and CNS drug levels. Participants in our current study may have been more aware of receiving MPH after completing both EEG sessions compared with our previous study (Tan & Hagoort, 2020), as participants' BL-VAS rating remained unchanged in the previous study. However, in line with our earlier findings, we did not find evidence linking MPH administration to changes in sEBR. This underscores the need for caution when using sEBR to approximate catecholamine activity (Tan & Hagoort, 2020; van den Bosch et al., 2023).

Supplementary Figure A. Marginal posterior probability distributions for the Drug-related effects in the Bayesian models designed to explore the Ambiguity, Drug, ROI and Drug Order effect in early N400, late N400, as well as P600 time windows time-locked to critical word. Solid vertical blue lines indicate the mean of the distribution and blue shaded region indicate 95% CrI. The drug-related predictors are presented in bold fonts. "Amb" represents the predictor for Ambiguity effect; "Ant" represents the predictor for Anteriority effects (Anterior vs. Posterior electrodes); "Hemi" represents the predictor for Hemisphere effects (Left vs. Right electrodes); “Ord” represents the predictor for Order effects (MPH-PLA vs. PLA-MPH). An asterisk (*) indicates results where zero falls outside the 95% CrI of the posterior estimates. A dot (·) indicates results where zero falls outside the 90% CrI of the posterior estimates.

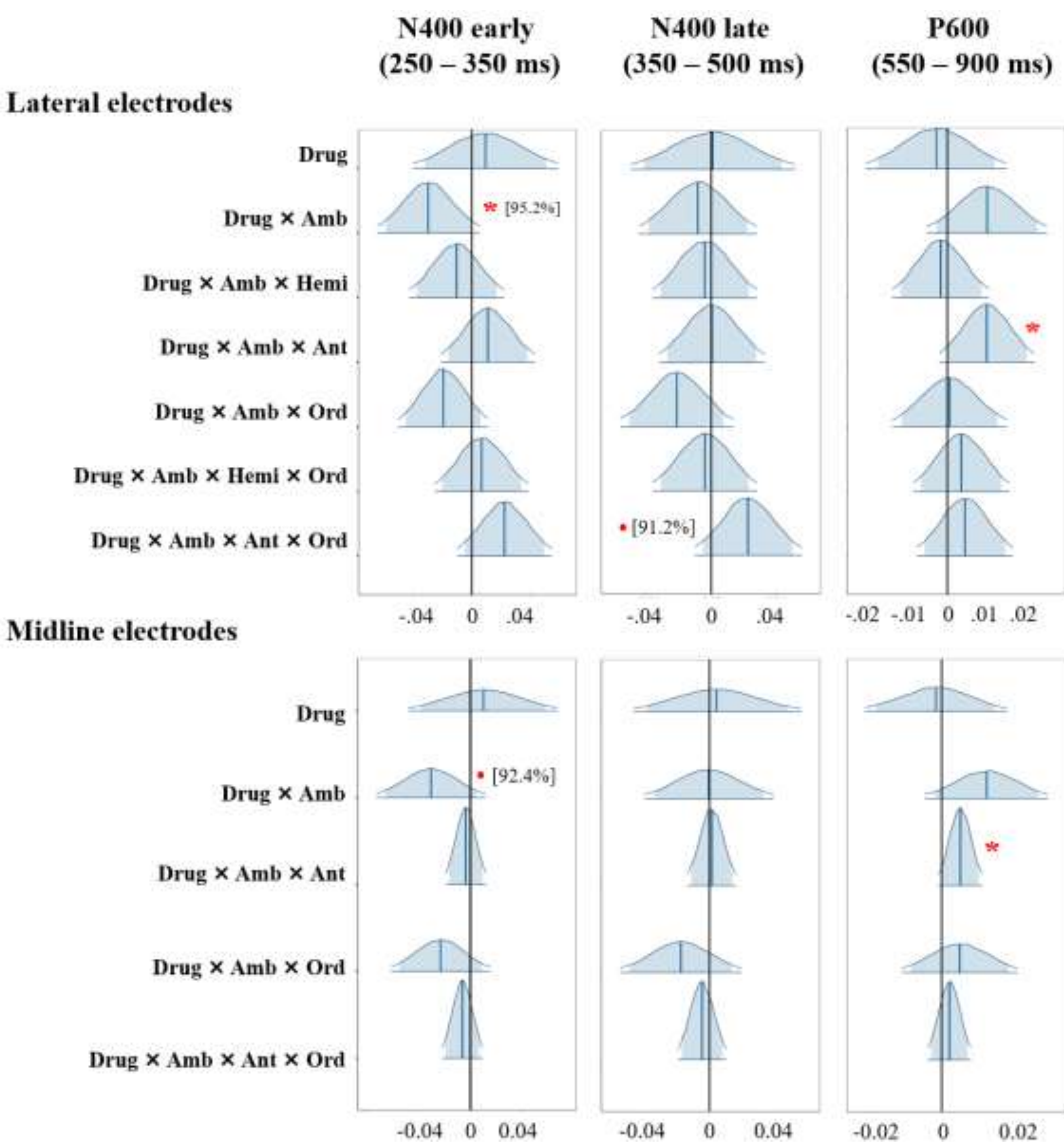

Supplementary Figure B. (a) Grand-averaged ERPs (n = 30) at nine representative electrodes time-locked to the second noun (e.g., “drummer”) in the Comma (Unambiguous) and No-Comma (Ambiguous) conditions, split into data from methylphenidate (MPH) and placebo (PLA) EEG sessions respectively. Negativity is plotted upwards. (b) Scalp distribution of the amplitude difference between data from the MPH and PLA sessions time-locked to the second noun. For illustrative purpose only, a 15-Hz low-pass filter and a baseline correction from -200 to 0 ms relative to critical word onset was applied. There was no significant CPS effect at the second noun (e.g., “*drummer*”). This aligns with Kerkhofs et al. (2008), who suggested that CPS may only become manifest when participants explicitly attend to punctuation. In our study, punctuation was relatively rare (16.8% of sentences) and error-free, making it unlikely that participants focused on it overtly. This may explain why, unlike in some silent reading studies (Steinhauer & Friederici, 2001), no CPS effect was observed.

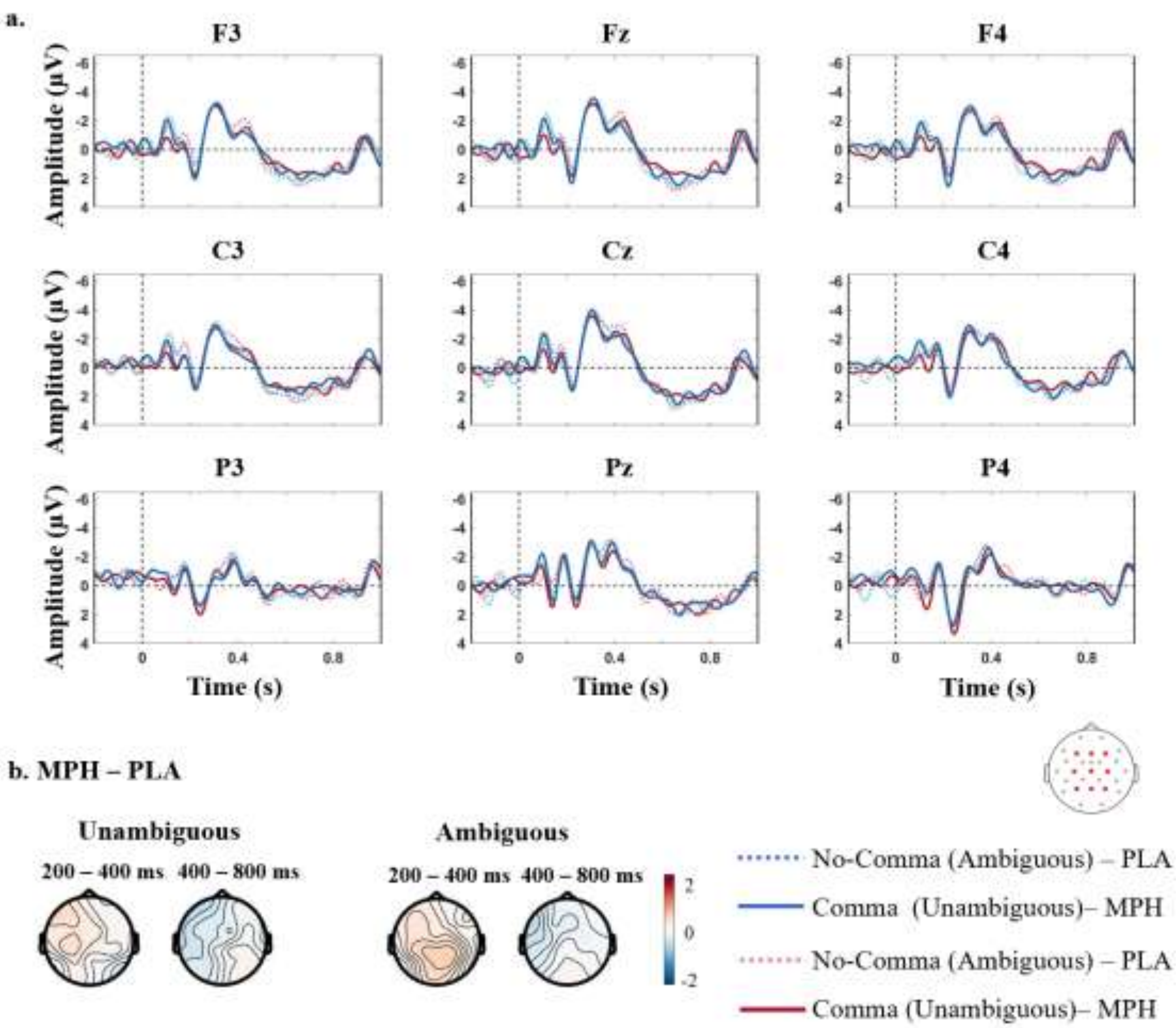

Supplementary Figure C. Marginal posterior probability distributions for the predictors of interests in the Bayesian models designed to explore the accuracy and RT data for the Ambiguity, Drug, and Drug Order effects. An asterisk (*) indicates results where zero falls outside the 95% CrI of the posterior estimates. A dot (·) indicates results where zero falls outside the 90% CrI of the posterior estimates.

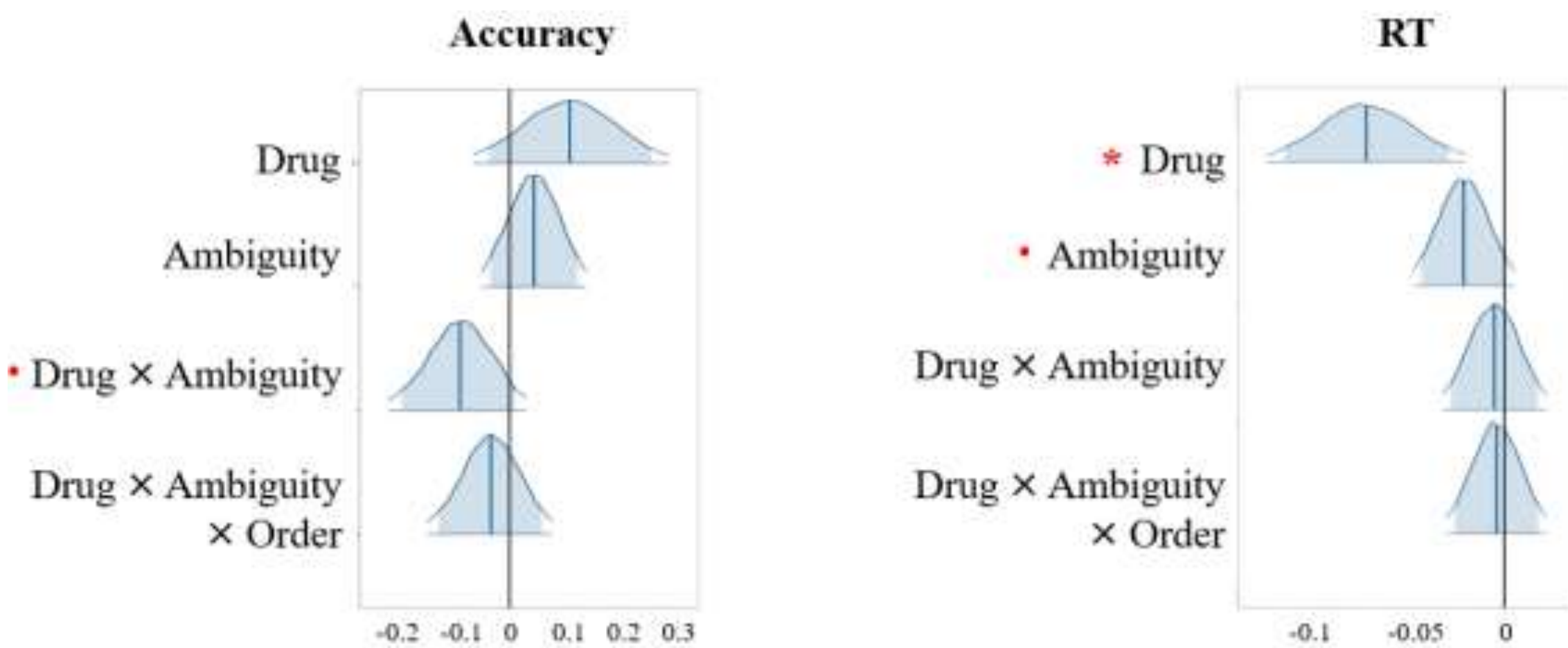

Supplementary Figure D. (a) Grand-averaged ERPs (n = 30) at nine representative electrodes (highlighted in red on the topographic plot), and (b) their topographical distribution time-locked to the onset of the critical word in the ambiguous and unambiguous conditions, separately for the MPH and Placebo (PLA) EEG sessions respectively. Negative values are plotted upwards. For illustrative purpose only, a 15-Hz low-pass filter has been applied to the waveforms, and a baseline correction applied from -200 to 0 ms relative to the onset of the critical word.

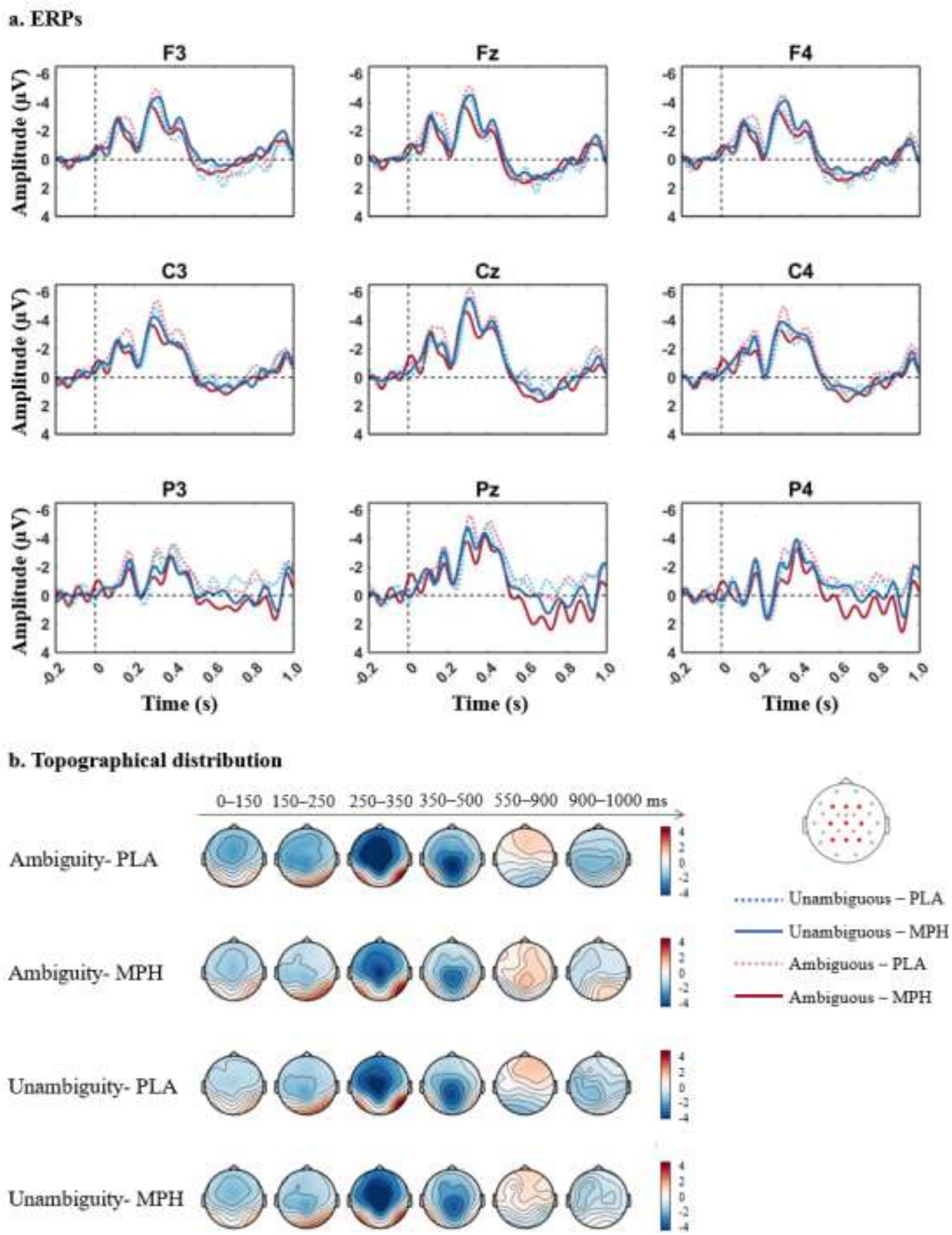

Supplement: Supplementary file 1 [file pnas.2606806123.sapp.pdf]
